# Authors’ Response to Peer Reviews of “The Performance of DeepSeek R1 and Gemini 3 in Complex Medical Scenarios: Comparative Study”

**DOI:** 10.2196/96220

**Published:** 2026-04-27

**Authors:** Maria Bajwa, Robert Hoyt, Dacre Knight, Maruf Haider

**Affiliations:** 1 MGH Institute of Health Professions, Boston, MA, United States; 2 Internal Medicine Department, Virginia Commonwealth University, 57 North 11th Street, Richmond, VA, 23298, United States, 1 8503845235; 3 Internal Medicine Department, University of Virginia, Charlottesville, VA, United States; 4 Internal Medicine Department, Carilion Roanoke Memorial Hospital, Roanoke, VA, United States

**Keywords:** large reasoning model, LRM, large language model, LLM, accuracy, medical scenario, DeepSeek R1, Gemini 3

*This is the authors’ response to the peer review of “The Performance of DeepSeek R1 and Gemini 3 in Complex Medical Scenarios: Comparative Study.”*

## Round 1 Review

### Reviewer B [1]

#### General Comments

*This is a timely and well-structured paper [2] that investigates the application of DeepSeek R1, a state-of-the-art large reasoning model, in the medical domain using the Massive Multitask Language Understanding Pro (MMLU-Pro) benchmark. This paper presents a follow-up evaluation of the DeepSeek R1 large reasoning model on open-ended medical scenarios from the MMLU-Pro benchmark. The study finds that DeepSeek R1 achieves a high accuracy of 92% without multiple-choice options, demonstrating its potential utility in more realistic clinical settings. The paper is timely and relevant, with strong empirical results and clear motivation. However, it would benefit from revisions to improve clarity, contextual grounding in existing work, and methodological detail. The authors may also consider citing recent work that examines the questioning strategies of large language models (LLMs) in clinical dialogues to better position this study in the broader landscape.*

**Response:** We appreciate the reviewer’s comment, and we have now added citations and improved the clarity and contextual grounding of the paper.

*The study is commendable for its effort in combining expert validation with benchmark testing and highlighting both performance and interpretability aspects. The paper is generally well-written, informative, and relevant to the research community on artificial intelligence (AI) in health care. However, before being suitable for publication, several important revisions are required. These include expanding the related work section to better situate the contribution of current research efforts, addressing some methodological limitations more transparently, and improving the robustness and generalizability.*

**Response:** We thank the reviewer for this comment and supportive critique. We have edited our conclusions and mentioned generalizability in the Discussion section. We have also combined the results of querying closed- and open-ended scenarios.

#### Major Comments

1. *While the paper references MMLU, MedQA, and some domain-specific LLM evaluations, it lacks a deeper discussion on recent approaches to questioning capabilities and long-context understanding in medical AI. Two notable papers should be included. First, “HealthQ: Unveiling Questioning Capabilities of LLM Chains in Healthcare Conversations” by Wang et al [3]. This paper presents a benchmarking framework focusing on the inquiry and elicitation capacity of LLM chains, which directly relates to the “reasoning” and prompt design aspects discussed here. Second, ”Context Clues: Evaluating Long Context Models for Clinical Prediction Tasks on EHR Data” by Wornow et al [4]. This study highlights how context windows and task framing affect LLM performance on clinical reasoning—relevant for understanding how question complexity and format might interact with LLM accuracy. The paper could be strengthened by referencing more recent work on prompting and questioning strategies in clinical LLM applications. The paper would benefit from referencing Wang et al [3], which evaluates LLM chains’ ability to optimize questions through reflection and prompting. This is relevant to the current paper’s interest in open-ended diagnostic reasoning and LLM behavior in clinical settings.*

**Response:** We thank the reviewer for providing this information and for highlighting these notable papers. We have now accordingly mentioned the important work done by these researchers and also cited these papers in the Introduction section.

*2. Although the DeepSeek R1 model is rigorously evaluated against MMLU-Pro, there’s a lack of direct performance comparison to other LLMs (eg, MedPaLM, GPT-4, Claude) on the same dataset or medical scenarios. Even informal or partial benchmarks would help contextualize the model’s effectiveness. Also, the novelty should be better emphasized—is this the first comprehensive large reasoning model evaluation on MMLU-Pro’s health subset?*

**Response:** This is the first comprehensive evaluation of a large reasoning model on the MMLU-Pro dataset. We acknowledge the comparative analysis gap with other LLMs. However, it was beyond the scope of this study. We have noted this in our Limitations section. In future studies, we plan to do a comparative analysis with multiple LLMs.

*3. The paper rightly points out issues with cueing and “testwiseness” in multiple-choice questions but doesn’t propose concrete mitigations. The planned future work of testing without answer choices is excellent—consider incorporating a small pilot of this now or discussing expected outcomes in more depth. Also, the limitations of using only 162 scenarios across many specialties could be made more transparent, especially regarding statistical robustness and specialty-specific insights.*

**Response:** The manuscript addresses the problem of cueing and testwiseness in multiple-choice questions by incorporating a second study phase in which open-ended prompts were used to remove answer choices to compare against testwiseness. While the influence of testwiseness remains to be fully quantified, our findings suggest that prompt format plays a significant role in model performance, underscoring the importance of further experimentation with different assessment designs. We also note the limited sample size as a study limitation, recognizing that the use of only 162 scenarios across numerous specialties restricts statistical robustness and the depth of specialty-specific insights. Future research is warranted to improve assessment and expand the dataset size for greater generalizability.

*4. The study uses a fixed prompt but does not explore or discuss the impact of prompt variations, which may influence results in open-ended tasks.*

**Response:** We agree with the reviewer. Prompt engineering should have been undertaken early in the research process.

*5. While the discussion of biases and failure modes is helpful, a more structured breakdown of error types and their frequency would improve the interpretability of findings.*

**Response:** This is a good point; we thank the reviewer for the suggestion. We have now added a discussion of error types and frequency.

*6. The discussion on reasoning steps and transparency is insightful but could be expanded to address recent concerns about the faithfulness of chain-of-thought outputs.*

**Response:** We thank the reviewer for this comment and have now expanded the discussion on reasoning steps and chain-of-thought outputs.

#### Minor Comments

*7. Model latency and usability: while the latency of DeepSeek is acknowledged, it’s not contextualized with respect to potential clinical utility or workflow integration. A brief paragraph on practical deployment implications would strengthen the discussion.*

**Response:** Latency of 15 to 20 seconds was experienced, and we concluded that this was inconsequential and did not any comment.

*8. Citation formatting: ensure all references (especially web-based ones like Perplexity and PromptHub) are consistently formatted and maintained in the reference list.*

**Response:** Agreed; these have been updated in the reference list.

*9. Future directions could be made more actionable by suggesting benchmark expansions with real patient data or multimodal inputs.*

**Response:** We agree; we have added a section on future implications, and we believe both of these to be viable (data and inputs).

### Reviewer Q [5]

#### General Comments

*This paper [2] seeks to evaluate the accuracy of DeepSeek R1 in correctly identifying the primary medical diagnosis in the medical scenarios dataset portion of MMLU-Pro using an open-ended format. Some clarifications on the methods and results (especially around the roles of subject matter experts vs core team members in the publication), would be helpful in understanding how these results were derived.*

**Response:** We have now added clarifications on the methods, and we thank the reviewer for making this suggestion to help the reader understand our results.

#### Minor Comments

*1. Introduction: consider citing Deepseek AI’s Deepseek R1 paper* [6].

**Response:** We thank the reviewer for these additional comments. We have now included the suggested reference, as we think it will improve the context of this study.

*2. Methods: please clarify who your subject matter experts were (eg, physicians, researchers) in terms of rank, specialty, and role and how they were used to grade answers (eg, selected based on specialty, 2 reviewer process, etc).*

*3. Methods: please indicate when the analyses were run.*

**Response:** In the current work, since we have merged 2 papers, we have excluded the subject matter expert opinions.

*4. Results: who determines whether references are related or unrelated?*

**Response:** References were assessed by the authors of this paper; we have now clarified this.

*5. Results and Discussion: it is unclear to me from reading the discussion portion of the paper as to whether we have any sense of whether DeepSeek R1 has correct reasoning for questions with correct diagnoses (eg, it may get the right diagnosis but may have incorrect reasoning). Similarly, did you determine the “correct answer” based on string matching (for example, if the answer was “septic arthritis” and the DeepSeek output stated “septic shock,” would this be incorrect)?*

**Response:** With regard to the discussion on verifying the reasoning, this is an excellent point. Unfortunately, within the scope of this current study, we were unable to assess specific reasoning steps beyond determining accuracy. String matching was not used for determining validity.

*6. Discussion: consider acknowledging the sample size of questions as a limitation.*

**Response:** We agree that the sample size was small, and we have now mentioned this as a limitation of this study.

### Reviewer AA [7]

#### General Comments

*This paper* [2] *reports on an experimental study to analyze the MMLU-Pro Q&A dataset. The authors find that DeepSeek R1 had an accuracy rate of 95.1% in 162 medical scenarios after reconciliation with subject matter experts on 23 questions. The findings contribute to the growing body of knowledge on LLM applications in health care and provide insights into the strengths and limitations of DeepSeek R1 in this domain.*

**Response:** We appreciate the reviewer’s comments, as this was our primary objective. In the current merged study, in order to use uniform formats (for both closed- and open-ended questions), we omitted the subject matter experts, removed the corresponding references and data, and recalculated the accuracy.

#### Major Comments

*1. The results are not appropriately qualified with results on statistical significance, and/or are lacking comparisons with other language models. Even if we know how other models perform overall, it would still be good to have more details, such as a comparison of where one model is right and another is wrong. Those kinds of deep insights are lacking in this paper. All we really know is that DeepSeek performs at a level roughly equivalent to the other leading models (nothing surprising there) and that it sometimes has incomplete or inexplicable behavior. I feel the paper needs to have more results and analysis to be a good fit for this journal.*

**Response:** Thank you for your comment. We have expanded the Results section by conducting quantitative and qualitative analysis. Conducting the analysis by using other models was beyond the scope of this study. This particular point has been listed in the Future Recommendations section. [**Editor’s note:** In response to the editor’s advice, the authors updated the analyses and final (accepted) version of the manuscript to include a comparison with another model (Gemini).]

*2. Maybe you could add a workflow diagram/figure to better illustrate the methods?*

**Response:** We have expanded the Methods section and and included the prompts used and an MMLU-Pro question and answer as an illustrative example. Therefore, we do not think adding a diagram would add value to the manuscript.

*3. I would like Table 1 to be augmented. Perhaps you can add an example question with answer choices? Right now, it looks very trivial. The alternative is to create a simple bar graph instead of a table, but the former would be more useful.*

**Response:** We have now removed this table from our updated, combined manuscript. We also have added an example question and associated answer choices to augment the methodological reporting of this study.

## Round 2 Review

### Reviewer B

*My comments have been addressed.*

**Response:** Thank you.

### Reviewer Q

*The paper has been revised to address the Transparent Reporting of a Multivariable Prediction Model for Individual Prognosis or Diagnosis–Large Language Model (TRIPOD-LLM) guidelines. Overall it appears most concerns from both reviewers have been addressed.*

**Response:** Thank you.

## Abbreviations

AI: artificial intelligence
LLM: large language model
MMLU-Pro: Measuring Massive Multitask Language Understanding Pro
TRIPOD-LLM: Transparent Reporting of a Multivariable Prediction Model for Individual Prognosis or Diagnosis–Large Language Model

## References

[1] Wang Z (2026). Peer review of “The Performance of DeepSeek R1 and Gemini 3 in Complex Medical Scenarios: Comparative Study". JMIRx Med.

[2] Bajwa M, Hoyt R, Knight D, Haider M (2026). The performance of DeepSeek R1 and Gemini 3 in complex medical scenarios: comparative study. JMIRx Med.

[3] Wang Z, Li H, Huang D, Kim HS, Shin CW, Rahmani AM (2025). HealthQ: unveiling questioning capabilities of LLM chains in healthcare conversations. Smart Health (2014).

[4] Wornow M, Bedi S, Hernandez MAF Context clues: evaluating long context models for clinical prediction tasks on EHR data.

[5] You JGT (2026). Peer review of “The Performance of DeepSeek R1 and Gemini 3 in Complex Medical Scenarios: Comparative Study. JMIRx Med.

[6] Guo D, Yang D, Zhang H (2025). DeepSeek-R1: incentivizing reasoning capability in llms via reinforcement learning. arXiv.

[7] Kejriwal M (2026). Peer review of “The Performance of DeepSeek R1 and Gemini 3 in Complex Medical Scenarios: Comparative Study. JMIRx Med.

